# Sustainable quality improvement interventions to ensure adequate physician documentation of chaperone presence in breast clinic: a multicycle clinical audit

**DOI:** 10.1093/intqhc/mzag141

**Published:** 2026-09-21

**Authors:** Cian M Hehir, Gavin G Calpin, Nadia Walsh, Gordon R Daly, Gavin P Dowling, Sandra Hembrecht, Katie Kearns, Michael Allen, Abeeda Butt, Colm Power, Arnold D K Hill

**Affiliations:** Department of Surgery, Royal College of Surgeons in Ireland, Dublin, Ireland; Department of Breast, Endocrine & General Surgery, Beaumont Hospital, Dublin, Ireland; Department of Surgery, Royal College of Surgeons in Ireland, Dublin, Ireland; Department of Breast, Endocrine & General Surgery, Beaumont Hospital, Dublin, Ireland; Department of Breast, Endocrine & General Surgery, Beaumont Hospital, Dublin, Ireland; Department of Surgery, Royal College of Surgeons in Ireland, Dublin, Ireland; Department of Breast, Endocrine & General Surgery, Beaumont Hospital, Dublin, Ireland; Department of Surgery, Royal College of Surgeons in Ireland, Dublin, Ireland; Department of Breast, Endocrine & General Surgery, Beaumont Hospital, Dublin, Ireland; Department of Surgery, Royal College of Surgeons in Ireland, Dublin, Ireland; Department of Breast, Endocrine & General Surgery, Beaumont Hospital, Dublin, Ireland; Department of Nursing, Beaumont Hospital, Dublin, Ireland; Department of Breast, Endocrine & General Surgery, Beaumont Hospital, Dublin, Ireland; Department of Breast, Endocrine & General Surgery, Beaumont Hospital, Dublin, Ireland; Department of Breast, Endocrine & General Surgery, Beaumont Hospital, Dublin, Ireland; Department of Surgery, Royal College of Surgeons in Ireland, Dublin, Ireland; Department of Breast, Endocrine & General Surgery, Beaumont Hospital, Dublin, Ireland

**Keywords:** chaperone, breast examination, outpatient clinic, quality improvement, audit, electronic patient records

## Abstract

**Introduction:**

Current best practice guidelines recommend chaperone presence during intimate clinical examination. Achieving guideline standards of documentation regarding chaperone presence has important implications from both clinical governance and medicolegal perspectives. This multicycle clinical audit provides insight into quality improvement interventions relevant to chaperone utilization and achieving guideline documentation standards.

**Materials and methods:**

This single-centre, multicycle quality improvement audit was performed in the setting of a tertiary breast referral clinic at Beaumont Hospital, Dublin, Ireland. The quality improvement audit consisted of three audit cycles with two distinct interventions employed. Firstly, a ‘chaperone stamp’ was entered into the clinical notes prior to consultation to serve as a memory aid for physicians. Secondly, following transfer of clinical notes from paper-based to electronic records, a mandatory check field was introduced ensuring adequate documentation.

**Results:**

A total of 388 female patients underwent breast examination at our centre. Despite levels of chaperone presence being directly observed at 100% in clinical consultations, chaperone documentation rates were poor (7.6%). Introduction of a chaperone stamp resulted in a 51.1% improvement in chaperone documentation. This intervention demonstrated poor sustainability as documentation standards declined by 13.6% at 6 months postintervention. Introduction of a mandatory check field using electronic patient records generated the most significant improvement in documentation standards, with 100% of clinical notes recording chaperone presence to reference standard.

**Conclusion:**

Accurate documentation of chaperone presence or decline thereof is of relevance from clinical governance and medicolegal perspectives. Simple, resource-efficient interventions such as the ‘chaperone stamp’ demonstrate significant quality improvement potential but with questionable long-term efficacy. Electronic records present a viable opportunity for units to achieve guideline standards in a time and resource-efficient manner.

## Introduction

Intimate examinations include the physical assessment of breasts, genitalia, and rectum. However, this is a non-exhaustive list, and patients’ perceptions of what may be deemed intimate must be taken into consideration [1, 2]. Guidelines on the use of a chaperone for intimate examinations are described by both the General Medical Council (GMC) and the Irish Medical Council (IMC) [1, 3]. These guidelines outline that a chaperone should be offered to all patients when performing intimate examinations [1, 3].

In general, a chaperone is a healthcare professional who is trained for the role and is familiar with the procedure to be observed [1]. Their presence should ensure respect to the patient’s dignity and confidentiality, and alert to the presence of distress or discomfort [1]. As highlighted by the Medical Protection Society and the IMC, a chaperone is to safeguard both the doctor and the patient, and his/her presence during examination should be recorded [2, 3].

The evidence base evaluating the effect of chaperone presence on patient experience is conflicting. A UK-based study, aimed at assessing patient preference regarding chaperone presence during breast examination, reported a majority (52%) of patients felt it was unnecessary, while 33% preferred to have one present [4]. Sixty-eight percent of the same cohort felt that offering of a chaperone was a sign of respect and posited that the ‘attitude’ and gender of the clinician had the most influence on whether they would prefer a chaperone present or not [4]. A randomized controlled trial evaluating patient experience with chaperone presence during proctological examination demonstrated that patients were generally happier to forego chaperone presence [5]. Current national and international guidelines are reflective of this—chaperone presence should be offered to all patients but is not a requirement [1, 3]. The most commonly cited reason for doctors preferring chaperone presence centres around potential medicolegal scrutiny [6]. As such, achieving documentation standards reflective of best practice guidelines is an essential step in clinical governance in the setting of intimate medical examination. Multiple groups have attempted to enhance documentation standards surrounding chaperone presence through implementation of quality improvement interventions [7–9].

We aimed to assess documentation rates of chaperone presence during breast examinations in our institution. We aimed to compare these results to established national guidelines and develop an intervention capable of enhancing departmental performance in a sustainable, patient-centric manner.

## Materials and methods

This quality improvement audit was performed in a single tertiary centre for symptomatic breast referrals—Beaumont Hospital, Dublin, Ireland. It was conducted in accordance with the audit cycle, which is a cyclical 6-stage process for implementing quality improvement outlined by the National Institute for Clinical Excellence (NICE).

The guidelines chosen were based on the aspects of care we wished to examine, prestated or implicit levels of success we wished to achieve, and targets or the defined level of expected compliance with our audit criteria. The chosen standard for comparison was that of the *Irish Medical Council’s Guide to Professional Conduct and Ethics for Registered Medical Practitioners, 8th Edition, Article 35.3,* which recommends the following:

1. All patients undergoing intimate examination should be offered the presence of a chaperone.
2. Offering of a chaperone should be documented in the patients’ notes.
3. If chaperone is requested by the patient, the identity of the chaperone should be recorded in the patient’s notes.
4. If chaperone presence is refused by the patient, this should also be documented.

The symptomatic breast clinic was prospectively selected as the study setting, given all patients attending undergo triple assessment, of which clinical breast examination forms a routine component. As the audit standard related specifically to documentation of chaperone presence during intimate examination, this setting provided a patient population uniformly eligible for assessment. Accordingly, inclusion criteria comprised all female patients attending the symptomatic breast clinic who had documented evidence of undergoing breast examination during the predefined audit periods. Patients who did not undergo breast examination and male patients were excluded.

This audit was prospectively registered with the clinical audit office at Beaumont Hospital (CA2023/178). Charts were retrospectively reviewed following completion of clinic with data logged manually by the research team using *Microsoft Excel*. During preintervention 1 and postintervention 1 audit cycles, a member of the research team was present in clinic to document the proportion of consultations in which a chaperone was present.

To ensure representation of all members of the breast surgery team, consultant-led clinics were sampled in rotation throughout each audit period. Following completion of each clinic, 10 eligible patient charts were selected at random for retrospective review and data extraction. This sampling methodology was applied consistently across all audit cycles, with the exception of audit cycle 3, in which electronic patient records were reviewed. The following variables were recorded in all audit cycles:

1. Chaperone presence in observed clinic consultations;
2. Chaperone presence documented in clinical notes or documentation of chaperone refusal;
3. Gender of clinician documenting the clinical encounter.

Preintervention data collection was commenced on 18 May 2023 for a predetermined period of time and concluded on 16 June 2023. Documentation standards were compared to the IMC guidelines, and results were analysed and disseminated to all staff members working in the breast clinic. Reasons for non-compliance with the guidelines were identified to assist in the development of a strategic intervention plan. Findings from each audit cycle were reviewed at departmental meetings and informed the development of subsequent interventions, allowing progressive refinement of the quality improvement interventions/strategy.

Three audit cycles were performed in total, which captured the data of *n* = 388 female patients. A temporal process map presents a schematic representation of our multiaudit cycle, including the timing, duration, and purpose of sampling as well as the process of iterative learning and intervention evolution (Fig. 1). During the relevant audit cycles, three discrete interventions were introduced:

**Figure 1:**
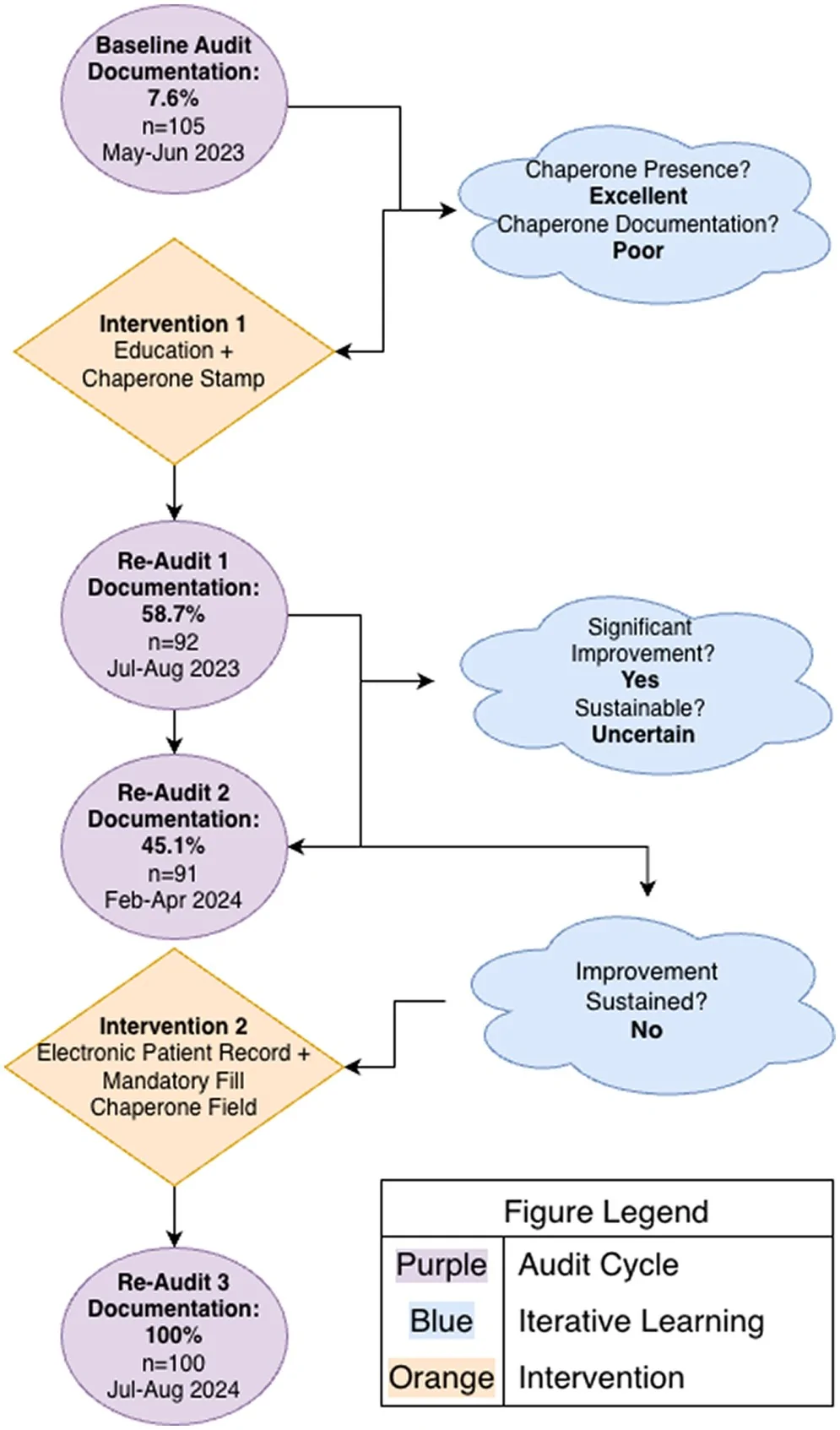
Temporal process map illustrating iterative learning and intervention evolution across successive audit cycles aimed at improving compliance with Irish Medical Council standards for documentation of chaperone presence during breast examination.

1. Research team member-led staff education—provision of team members with Irish Medical Council guidelines surrounding chaperone presence and documentation (written and verbal). Physicians’ understanding of guidelines and their practical applications was discussed in an open-forum discussion amongst team members. Between audit cycles, the results were discussed in an open forum, and team members were invited to discuss strategies to further improve adherence.
2. Introduction of a chaperone documentation stamp to patient files.
3. Development of an electronic consultation system with mandatory recording of chaperone presence or reason for chaperone absence.

The timing, duration, purpose, and sample size of each have been summarized for ease of interpretation (Table 1). Detailed information of the audit cycles, the manner in which they were conducted, their purpose, and the evolution of each proposed intervention are successively outlined below.

**Table 1:** Summary table of audit cycle timing, duration, purpose, and sample size evaluated across successive audit cycles evaluating compliance with chaperone documentation standards in a symptomatic breast clinic.

Audit cycle	Dates	Duration (weeks)	Purpose	*n*
Baseline audit	18 May–16 June 2023	4	Baseline assessment of documentation practices	105
Re-audit 1	24 July–18 August 2023	4	Short-term assessment following education and chaperone stamp introduction	92
Re-audit 2	5 February–12 April 2024	10	Sustainability assessment of Intervention 1	91
Re-audit 3	July 2024	10	Assessment following EHR mandatory field implementation	100

### Intervention 1—Staff education and introduction of a chaperone stamp

Following collection and interpretation of preintervention audit, results were shared at the weekly departmental meeting. Education regarding the current national guidelines on chaperone presence for intimate examination as well as the documentation requirements for the same was carried out. The breast surgery department identified the use of a chaperone stamp as a potential means for clinical quality improvement. This stamp was prospectively entered into the patient chart prior to clinical consultation to act as a reminder to clinicians.

### Postintervention 1—Re-audit

The impact of the chaperone stamp intervention was evaluated on the second audit loop conducted between 24 July 2023 and 18 August 2023. Following the introduction of the chaperone stamp, chaperone documentation compliance was re-audited on two occasions, at (i) 6 weeks and (ii) 6 months postintervention. The 6-month re-audit data collection period involved collecting documentation rates from 10 patient charts over a 10-week period, commencing on 5 February 2024 and concluding on 12 April 2024. The rationale for re-auditing at two disparate time intervals was to assess the long-term feasibility of stamp introduction as a quality improvement intervention (Fig. 1).

### Intervention 2—Electronic consultation records and mandatory chaperone field

Throughout the second re-audit cycle, our department was in the process of introducing electronic consultation records to outpatient breast consultations. Information technology (IT) colleagues developed a mandatory chaperone field which would not permit submission of the consultation document to the patient record without:

1. Documentation of chaperone being offered.
2. If chaperone accepted—identity of chaperone.
3. If chaperone declined—reason why.

### Postintervention 2—Re-audit

Electronic clinic records were retrospectively screened. In July 2024, a random sample (*n* = 100) of the most recent consultation notes were reviewed. Data were manually transferred to *Microsoft Excel* for analysis.

Between audit cycles, results were presented at departmental meetings with education re-iterated regarding the current guidelines surrounding chaperone presence and documentation during intimate examinations.

### Statistical analysis

Raw data were imported and analysed using R Statistical Software [10]. A two-tailed Fisher’s exact test was employed to determine whether there were statistically significant differences between groups of categorical variables.

## Results

Throughout this multicycle clinical audit, the clinical notes of 388 patients were retrospectively reviewed following consultation and subsequent clinical examination at a symptomatic breast tertiary referral centre. This sum can be further divided into preintervention (*n* = 105), postintervention 1 re-audit cycle 1 (*n* = 92), postintervention 1 re-audit cycle 2 (*n* = 91), and postintervention 2 re-audit cycle 1 (*n* = 100) (Table 2).

**Table 2:** Summary of the documentation of chaperone presence throughout the multicycle audit.

	Clinician gender	Preintervention audit		Postintervention 1 Cycle 1	Postintervention 1 Cycle 2		Postintervention 2 Cycle 1
Total consultations audited		105	INT^a^ - 1 Education and chaperone stamp	92	91	INT - 2 Electronic patient record	100
Chaperone presence observed		(100%)		(100%)	–		–
Guideline standard documentation surrounding chaperone presence	Male	8/49 (16.3%)		48/70 (68.6%)	25/40 (62.5%)		64/64 (100%)
	Female	0/56 (0%)		6/22 (27.3%)	16/51 (31.37%)		46/46 (100%)
	Total	8/105 (7.6%)		64/92 (58.7%)	41/91 (45.1%)		100/100 (100%)

a INT, intervention.

During preintervention and postintervention 1 audit cycles, a member of the audit team directly observed all consultations and found chaperones to be present in 100% of consultations which required intimate examination. However, documentation of chaperone presence was poorly reflective of this finding, as indicated by the preintervention documentation rate of 7.6%. Notably, documentation of chaperone presence/patient refusal of chaperone presence varied significantly between male and female clinicians across all pre-electronic record audit cycles (Table 3). Throughout our audit cycles, male clinicians were significantly more likely to record chaperone details when performing breast examinations on female patients (*P* < .05) (Table 3).

**Table 3:** Relationship between gender and documentation of chaperone presence/patient refusal of chaperone presence^a^.

		Documented	Not documented	Total	*P*
Preintervention audit	Male doctors	8	41	49	*P* = .003*
	Female doctors	0	56	56	
	Total	8	97	105	
Postintervention 1 Re-audit 1	Male doctors	48	22	70	*P* = .003*
	Female doctors	6	16	22	
	Total	54	38	92	
Postintervention 1 Re-audit 2	Male doctors	25	15	40	*P* = .002*
	Female doctors	16	35	51	
	Total	41	50	91	

a Evaluated using a two-tailed Fisher’s exact test. Male clinicians were significantly more likely to record chaperone details when performing intimate examinations on female patients.

* *P *< .05 = statistically significant.

The initial intervention of staff education yielded a statistically significant improvement in chaperone documentation (*P* < .0001). However, although increasing documentation by 51.1%, this improvement still fell significantly short of the desired standard of 100% compliance to IMC guidelines. Furthermore, interval re-audit of compliance demonstrated decline in efficacy of this intervention, with 58.7% and 45.1% documentation compliance at 6 weeks and 6 months postintervention, respectively. The decline in documentation standards was statistically significant (*P* < .0002).

The introduction of electronic consultation records resulted in the most significant improvement in chaperone documentation standards. Following the introduction of a mandatory chaperone field, 100% compliance with IMC guidelines was achieved.

## Discussion

### Statement of principal findings

Over a 2-year period, our department completed three audit cycles which demonstrated significant improvement in physician documentation surrounding the use of chaperone. Preintervention documentation levels were exceedingly poor at 7.6%. Initially, our team demonstrated the efficacy of a simple, resource- and time-efficient intervention—the chaperone stamp. Utilization of a chaperone stamp has been previously demonstrated as an effective quality improvement intervention in chaperone documentation in breast surgery clinic [9]. However, long-term sustainability of achieved standards following quality improvement intervention in healthcare has been previously demonstrated as poor [11]. Our group’s findings further support this clinical reality, as was demonstrated by the significant decrease in documentation between 6 weeks and 6 months following our chaperone stamp intervention, from 58.7% to 45.1%, *P* = .0002. This finding supports the necessity of interval re-audit as has been previously posited in the literature [12].

### Strengths and limitations

This study presents a robust assessment of clinical documentation practices relevant to chaperone utilization in breast clinic, with broader applicability to any intimate clinical examination setting. A key strength of this study is that it encompasses multiple closed-loop audit cycles. These audit cycles were carried out over a 2-year time period, capturing the documentation practices of multiple non-consultant hospital doctors as they rotated through the service. We have outlined key observations which strengthen the study’s findings, such as the significant difference in physician documentation practices when the examination is being performed by a same-sex physician.

This study was subject to several limitations. Firstly, the retrospective nature of the data collection introduces potential selection bias. Secondly, the data presented by our group is representative of physician documentation following breast examination of female patients in the setting of an outpatient breast clinic. Documentation standards may differ in in-patient settings and during other forms of intimate clinical examination. The generalizability of these findings to other intimate clinical examination settings is questionable, and should be interpreted with caution, with particular emphasis placed on the single organ (breast), gender (female), and setting (specialist outpatient clinic) to which these data pertain. The inter-gender differences in documentation standards reported amongst practicing physicians in our unit were of interest, but there is likely some effect of co-variables such as physician seniority (consultant vs trainee), number of years in training, and the clinic setting (initial vs follow-up appointment) which were not explored in this study and would benefit from further research.

Although all audit cycles were conducted within the same clinical environment, using homogeneous eligibility criteria, audit standards and a consistent sampling methodology, there remains potential for residual, unexplored confounding factors within these data. Variations in clinical workload and clinician staffing may have influenced the standard of documentation practice but were not formally evaluated in this research output. As this study was designed in accordance with the NICE clinical audit framework rather than a controlled interventional study, these factors were not formally measured or adjusted for. However, several design-based measures were employed from the outset to minimize variability, including the use of a single clinical setting, standardized audit criteria and rotational sampling of consultant-led clinics.

### Interpretation within the context of the wider literature

This article presents quality improvement insights with clinical relevance to physicians working within breast surgery, or any medical field which requires intimate patient examination. The importance of chaperone availability for patients undergoing intimate clinical examination has been highlighted internationally through the provision of best practice guidelines [1, 3]. It is critical to discern that such guidelines do not render chaperone presence mandatory during such clinical interactions but underscore the importance of physicians formally offering chaperone presence and having a chaperone readily available for patients who wish to avail of one. Patient attitudes toward chaperones seem reflective of this stance. Whitford *et al.* [13] reported that only 3% of males and 11% of females would prefer a chaperone to be present during intimate examination when their treating physician is of the same gender. Patient–physician gender difference is a reportedly significant predictor of patient preference for chaperone, 11% of males and 51% of females in the same study cohort reported preference for chaperone presence when their examining physician was of the opposite sex (*P* < .001) [13]. Interestingly, 90% of the female patients and 78% of the male patients surveyed felt it was important for a chaperone to be offered by treating clinicians [13]. Our audit findings are in line with the published literature, as male physicians in our unit were significantly more likely to achieve guideline documentation standards regarding chaperone presence (*P* < .05). The role of the chaperone is also of evolving importance for treating clinicians, however, given the global rise in medicolegal cases surrounding intimate examination [6, 14]. This article echoes many of the early findings reported by Rose *et al.* whilst generating additional insights with regard to the role of physician gender, to the sustainability of the ‘chaperone stamp’ based interventions and to the evolving role of electronic patient records [9].

### Implications for policy, practice, and research

If quality improvement interventions are ineffective at eliciting sustainable change, it is perhaps the nature of the intervention that is at fault. An ideal intervention should be focused on targeted process integration into workflow in a manner which is both time and resource efficient. This sentiment is especially relevant in the clinical setting where both time and resources must be safeguarded. The evolution of electronic health records (EHRs) presents an ideal opportunity to enact sustainable change through mandatory field processes as was employed by our unit. Following integration of EHR to our outpatient breast clinic, 100% compliance with target guidelines was achieved. This study demonstrates the capacity of EHR to facilitate quality improvement in healthcare. Similar findings have been reported across a range of medical settings, with EHRs demonstrating superiority with respect to documentation standards and time to documentation completion [15]. The introduction of mandatory-fill fields using EHRs has demonstrated promising advantages in several clinical areas, including physician documentation standards and optimizing paediatric immunization rates [15, 16]. Electronic does not necessarily imply superiority however, and some authors have cautioned units to avoid overutilization of mandatory fill-fields in attempts to limit the probability of ‘check-list fatigue’ and to prioritize documentation of clinical elements which have direct benefit to patient outcomes and/or experience [17]. The introduction of EHRs has brought with it the promise of improved healthcare quality, with postintroduction hospital metrics demonstrating significantly reduced documentation times, higher levels of guideline adherence with lower numbers of reported medication errors in most units [18]. However, these findings are not ubiquitous, highlighting the need for continuous focus on the design and implementation of successful systems in the quality improvement literature [18]. This article highlights the potential role for EHR-based quality improvement interventions but highlights that implementation of such should be judicious.

Clinical audit has maintained its place as a pillar of quality improvement in healthcare and has been identified as a pillar of clinical governance [19–21]. Arguably, the potential impact of many clinical improvement audits is diminished due to the lack of reporting of audit processes and outcomes in the literature. Our team aspires that, through sharing the details of interventions relevant to enhancing clinical performance, units may collaborate toward better, more sustainable quality improvement. This article aims to disseminate our findings regarding the use of documentation aids such as the chaperone stamp. We highlight the effect of such simple, inexpensive interventions in the short term but question their ability to generate sustainable quality improvement. Finally, we highlight the emerging role of IT and the electronic patient record in ensuring effective, sustainable quality improvement interventions.

## Conclusion

The presence of a chaperone during intimate examination is of importance to both patients and physicians alike. Documentation of chaperone presence/patient decline of chaperone presence presents an important clinical standard from a medicolegal perspective. This multicycle quality improvement study demonstrates that the utility of a specified chaperone stamp improves documentation rates but may not result in sustainable quality improvement. In our study, the introduction of electronic healthcare records and the incorporation of mandatory fields therein resulted in documentation standards reaching those of national and international guidelines.

## Data Availability

The data supporting the findings of this clinical audit are available within the hospital’s secure audit database and are not publicly available due to patient confidentiality. De-identified data may be made available from the corresponding author (C.M.H.) upon reasonable request and with appropriate institutional approval.

## Patient and public involvement statement

Patient and public involvement was not sought for the completion of these works.
